# *SETBP1* and miR_4319 dysregulation in primary myelofibrosis progression to acute myeloid leukemia

**DOI:** 10.1186/1756-8722-5-48

**Published:** 2012-08-08

**Authors:** Francesco Albano, Luisa Anelli, Antonella Zagaria, Nicoletta Coccaro, Paola Casieri, Angela Minervini, Giorgina Specchia

**Affiliations:** 1 Department of Emergency and Organ Transplantation (D.E.T.O.) - Hematology Section, University of Bari, 70124, Bari, Italy; 2 Hematology, Azienda Ospedaliera Universitaria Policlinico, P.zza G. Cesare, 11 70124, Bari, Italy

**Keywords:** Primary myelofibrosis progression, T(12;18)(p13;q12) translocation, *SETBP1* and miR_4319 dysregulation, Intronic miRNA

## Abstract

The molecular pathogenesis underlying the primary myelofibrosis (PMF) progression to acute myeloid leukemia (AML) is still not well defined. The involvement of microRNA (miRNA) is actually helping to shed light on an important issue in the occurrence of myeloproliferative neoplasms (MPNs). However, the role of intronic miRNA, derived from the intron regions of gene transcripts, has never been reported in MPNs. In this study, we describe a PMF case evolved to AML with a t(12;18)(p13;q12) rearrangement showing the downregulation of the intronic miR_4319 and the overexpression of its host gene, SET binding protein (*SETBP1*). A possible molecular mechanism regulating the PMF progression to AML is discussed.

## Letters to the editor

Primary myelofibrosis (PMF) is a clonal stem cell disorder currently classified as a myeloproliferative neoplasm (MPN). The primary disease-causing mutation in PMF is unknown. Janus kinase 2 (*JAK2*) gain of function (*JAK2V617F*) and *MPL* gene mutation have been described in approximately 50% and 5%–10% of PMF patients, respectively [1]. Recently, the involvement of microRNA (miRNA) has been reported in PMF, showing a positive correlation with *JAK2V617F* allele burden [2-4]. miRNA are a class of single-stranded noncoding RNA that bind to the 3'-untranslated region of target mRNA and suppress gene expression by translation repression or mRNA degradation. About 37% of miRNA appear to be located within introns of protein-coding genes and may be transcribed by their own promoters or by the host gene promoter with a correlated expression [5]. Many miRNA target genes show expression patterns that are significantly correlated with the expression of the miRNA host genes [5].

We describe here a PMF case evolving to acute myeloid leukemia (AML) with a t(12;18)(p13;q12) rearrangement, showing *SETBP1* overexpression and downregulation of intronic miR_4319. The expression dysregulation increased during PMF progression, suggesting the involvement of a novel molecular mechanism in the leukemogenesis.

## Case presentation

A 75-year-old man came to our attention in July 2008 with symptoms of anemia, leukocytosis, and splenomegaly. Peripheral blood analysis showed leukoerythroblastosis and a bone marrow biopsy revealed a megakaryocyte proliferation with atypia, together with reticulin fibrosis. Conventional cytogenetic analysis showed the following karyotype: 46,XY[20]. Molecular analysis excluded the presence of the 5*'BCR/3'ABL* transcript whereas *JAK2V617F* was detected. In accordance with World Health Organization (WHO) 2008 criteria, a diagnosis of PMF was made. Treatment with hydroxyurea was started. In August 2009, the patient showed a raised leukocytes count. A bone marrow aspirate detected 30% of the myeloid blasts, giving rise to the diagnosis of AML. Cytogenetic analysis revealed the following karyotype: 46,XY,t(12;18)(p13;q12)[20]. Molecular analysis demonstrated the presence of the *JAK2* wild-type gene. Treatment with low dose ARA-C was started but the patient died a few days later of disease progression.

**Figure 1:**
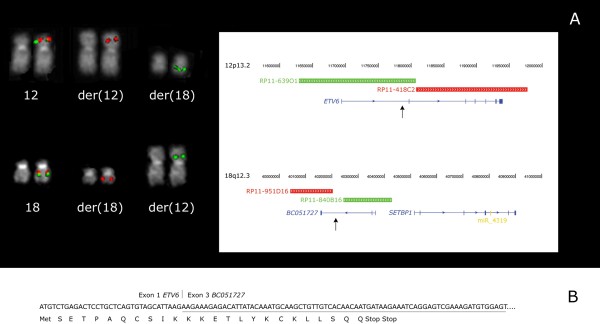
**FISH and molecular characterization of the t(12;18)(p13.2;q12.3) rearrangement. (A)** FISH analysis showing chromosome 12 and 18 breakpoints identification in the follow-up sample (on the left). A schematic representation of genes and BAC clones used in molecular cytogenetic experiments is shown (on the right). **(B)** RT-PCR experiments showing *5'ETV6/3'BCO51727* produced by the fusion of *ETV6* exon 1 with *BC051727* exon 3 (underlined). A truncated fusion protein was generated.

Fluorescence *in situ* hybridization (FISH) analysis was performed at the onset of the AML diagnosis using bacterial artificial chromosome (BAC) probes selected according to the University of California Santa Cruz (UCSC) database (http://genome.ucsc.edu/index.html; February 2009 release). This analysis showed the occurrence of a t(12;18)(p13.2;q12.3) rearrangement with breakpoints mapping inside *ETV6* on chromosome 12 and inside *BCO51727* on chromosome 18 (Figure 1A, B). Notably, the chromosome 12 breakpoint was mapped between the BAC clones RP11-639O1 and RP11-418C2 (12p13.2), whereas the clones RP11-951D16 and RP11-840B16 (18q12.3) delimited the breakpoint region on chromosome 18 (Figure 1A). At the PMF onset, FISH did not reveal the presence of the t(12;18) rearrangement.

Reverse transcription PCR (RT-PCR) experiments performed to verify the occurrence of possible fusion genes involving *ETV6* and *BCO51727* showed a *5'ETV6/3'BCO51727* fusion transcript using primers mapping inside *ETV6* exon 1 (5'GGGAGAGATGCTGGAAGAAACT3') and *BCO51727* exon 4 (5'AAGCCCAATGTTTCAAGACCTC3'). The *5'ETV6/3'BCO51727* fusion gene was shown to be constituted by *ETV6* exon 1 joined to *BCO51727* exons 3, 4, and 5 (Figure 1B).

**Figure 2:**
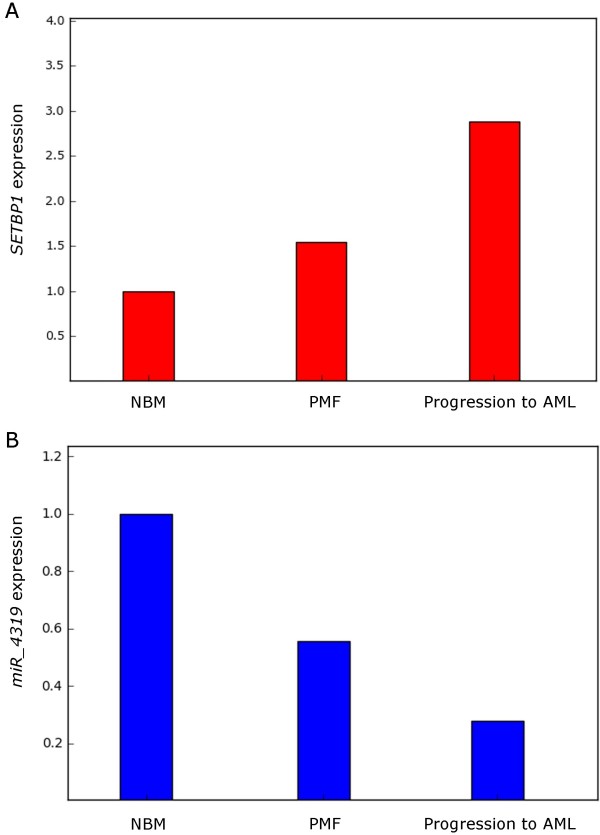
**Results of qRT-PCR studies. Graphic representation of *SETBP1 *(A) and miR_4319 (B) expression detected in the onset and follow-up samples of the analyzed patient and in normal bone marrow samples (NBM).**

This breakpoint was different from the one previously reported, but a truncated protein was produced resulting from the presence of premature stop codons (Figure 1B). No reciprocal *5'BCO51727/3'ETV6* fusion transcript was detected (data not shown). As the t(12;18) breakpoints were located about 300 kbp centromerically to *SETBP1* (18q12.3), we analyzed the gene expression by quantitative real-time PCR (qRT-PCR) experiments. The β-glucuronidase (*β-GUS*) gene was selected as an endogenous control and a pool of cDNA derived from bone marrow cells of ten healthy individuals was used as calibrator. This analysis showed *SETBP1* overexpression by a mean factor of 1.54 and 2.88 in samples at the onset of PMF and at progression to AML, respectively (Figure 2A).

The UCSC database was queried at the “sno/miRNA” track to verify the presence of miRNA close to the t(12;18) breakpoints; interestingly, miR_4319 mapped inside intron 4 of *SETBP1*. miRNA expression analysis was performed by qRT-PCR with a specific primer pair, Hs miR-4319 miScript Primer Assay, and RNU6B miScript Primer Assay for normalization (Qiagen). qRT-PCR experiments showed downregulation of miR_4319 by a mean factor of 0.35 and 0.18 at PMF onset and progression to AML, respectively (Figure 2B). A query of the MicroCosm (http://www.ebi.ac.uk/enright-srv/microcosm/) and TargetScan (http://www.targetscan.org/) databases did not elicit any predicted target genes of miR_4319.

## Conclusions

We report, for the first time, concomitant dysregulation of *SETBP1* and of its intronic miRNA in a PMF case evolving to AML showing a t(12;18)(p13.2;q12.3) rearrangement. To date, *SETBP1* overexpression has been described as a recurrent molecular event in 27.6% of AML patients at diagnosis, playing a crucial role in the leukemic transformation through the protein phosphatase 2A inhibition [6,7]. By contrast, the involvement of miR_4319 has never been previously demonstrated in AML.

It is known that intronic miRNA and host genes could show a correlative or discordant expression [5]. Several possible mechanisms could explain host-gene-independent expression, such as different transcript stabilities, post-transcriptional miRNA regulation, or the presence of a specific miRNA promoter [5,8]. In our case, the unrelated expression and opposite orientation between miR_4319 and *SETBP1* suggest the existence of different, independent promoters.

Moreover, the occurrence of *SETBP1* and miR_4319 dysregulation in the sample at diagnosis suggests the presence of the leukemic clone at too low a level to be detected by FISH. The disappearance during PMF progression of *JAK2V617F* at the same time as the leukemic clone expansion suggests an outstanding pathogenetic role for *SETBP1* and miR_4319 in PMF evolution to AML.

## Abbreviations

PMF: Primary myelofibrosis; AML: Acute myeloid leukemia; miRNA: MicroRNA; MPN: Myeloproliferative neoplasm; WHO: World health organization; FISH: Fluorescence *In Situ* Hybridization; BAC: Bacterial artificial chromosome; UCSC: University of California Santa Cruz; RT-PCR: Reverse transcription PCR; qRT-PCR: Quantitative Real-Time PCR; *β-GUS*: β-glucuronidase.

## Competing interests

The authors declare that they have no competing interests.

## Authors’ contributions

FA, LA, and AZ were involved in the design and execution of the experiments, wrote the manuscript and contributed to the overall experimental design. NC conducted FISH experiments. PC performed conventional cytogenetic analysis. AM contributed to perform molecular analysis experiments. GS supervised the manuscript preparation. All authors read and approved the final manuscript.
